# Does Direct MRI Tenography Improve the Diagnostic Performance of Low-Field MRI to Identify Artificially Created Soft-Tissue Lesions within the Equine Cadaveric Digital Flexor Tendon Sheath?

**DOI:** 10.3390/ani13243772

**Published:** 2023-12-07

**Authors:** Anton Aßmann, Stefanie Ohlerth, Silvana Hartmann, Paul Torgerson, Andrea Bischofberger

**Affiliations:** 1 Equine Hospital, Vetsuisse-Faculty, University of Zürich, 8057 Zürich, Switzerland; 2 Clinic of Diagnostic Imaging, Vetsuisse-Faculty, University of Zürich, 8057 Zürich, Switzerland; 3 Section of Veterinary Epidemiology, Vetsuisse-Faculty, University of Zürich, 8057 Zürich, Switzerland

**Keywords:** digital flexor tendon sheath, low-field MRI, tenography, gadolinium, deep digital flexor tendon, superficial digital flexor tendon, manica flexoria, proximal scutum, horse

## Abstract

**Simple Summary:**

Tenosynovitis of the digital flexor tendon sheath (DFTS), developing secondary to intrathecal tendinopathy, is commonly diagnosed using ultrasonography and contrast tenography. However, precise pre-operative diagnosis remains challenging. The objective of this study was to determine and compare the sensitivity and specificity of low-field MRI and direct low-field MRI tenography (MRIt) to detect experimentally created lesions of the superficial digital flexor tendon (SDFT), deep digital flexor tendon (DDFT), manica flexoria (MF) and proximal scutum in 21 DFTS cadavers. Proximal scutum lesions (Sensitivity: 38% versus 50%, *p* = 0.80; specificity: 96% versus 96%, *p* = 1) and SDFT lesions (Sensitivity: 39% versus 54%, *p* = 0.72; specificity: 93% versus 96%, *p* = 1) were less frequently identified by MRI compared to MRIt, respectively. MRI detected DDFT lesions (sensitivity 34%; specificity 100%) better than MRIt (sensitivity 32%, *p* = 0.77; specificity 98%, *p* = 1). This was similar for MF lesions (MRI sensitivity 61%; specificity 100% vs. MRIt sensitivity 50%, *p* = 0.68; specificity 96%, *p* = 1). Lesion size was significantly associated with MRI or MRIt diagnosis (*p* = 0.001).

**Abstract:**

Tenosynovitis of the digital flexor tendon sheath (DFTS) is diagnosed using ultrasonography and contrast tenography. Nevertheless, making a precise preoperative diagnosis is challenging. This study aimed to determine and compare the sensitivity and specificity of low-field MRI and MRI tenography (MRIt) to detect artificially created soft-tissue lesions in the DFTS. In 21 DFTSs, 118 lesions were made tenoscopically in the superficial digital flexor tendon (SDFT), deep digital flexor tendon (DDFT), manica flexoria (MF) and proximal scutum. MRI and MRI, following intrathecal gadolinium administration (MRIt), were performed. The sensitivity and specificity of MRI and MRIt were calculated and compared. Proximal scutum lesions were less frequently identified by MRI (Sensitivity 38%, specificity 96%) compared to MRIt (Sensitivity: 50%, *p* = 0.80; specificity: 96%, *p* = 1). This was similar for SDFT lesions (Sensitivity: 39% versus 54%, *p* = 0.72; specificity: 93% versus 96%, *p* = 1). MRI detected DDFT lesions (sensitivity 34%; specificity 100%) better than MRIt (sensitivity 32%, *p* = 0.77; specificity 98%, *p* = 1). This was similar for MF lesions (MRI sensitivity 61%; specificity 100% vs. MRIt sensitivity 50%, *p* = 0.68; specificity 96%, *p* = 1). Lesion size was significantly associated with MRI or MRIt diagnosis (*p* = 0.001). The intrathecal administration of gadolinium did not significantly improve the ability of low-field MRI to diagnose artificial DFTS tendon lesions. Small lesion length was a significant discriminating factor for lesion detection. MRI and MRIt specificity were high, thus being helpful in diagnosing an intact structure.

## 1. Introduction

Lameness attributed to digital flexor tendon sheath (DFTS) pathologies are common in horses. Moreover, DFTS tenosynovitis most commonly develops secondary to intrathecal tendinopathy. Overall, laterally situated marginal tears of the deep digital flexor tendon (DDFT) and manica flexoria (MF) tears belong to the most commonly reported intrathecal lesions [1,2,3].

Making an accurate preoperative diagnosis of these types of lesions remains challenging. Following the localization of lameness, DFTS ultrasonography is used as a first-line diagnostic tool; however, it has shown unreliable results for the detection of intrathecal tendinopathies [1,2,3,4,5]. The sensitivity of ultrasonographic lesion detection was in a low- to mid-range for MF tears (38%) and DDF tendinopathies (63% [1]–71% [3]). Furthermore, it could be shown that ultrasonography only detected lesions in up to 54% of the cases, when lesions were present tenoscopically [2,3]. Different non-weight-bearing, dynamic and dynamic synovial flow techniques have been described to improve the ultrasonographic diagnosis of MF tears [6,7].

Contrast radiography yielded more promising results compared to ultrasonography. Particularly for MF tears, higher sensitivity (92–96%) and specificity values (56–80%) are reported [8,9]. The results for DDFT lesions are lower with a sensitivity and specificity of 54% [8]–57% [9], and 73% [8]–84% [9], respectively. Nevertheless, in many cases, tenoscopy remains the diagnostic tool of choice for DFTS disorders, not only allowing for the visualization of a specific soft-tissue lesion but also its surgical debridement [3,4,5,10]. However, it would be desirable to have a standing diagnostic imaging tool that reliably detects these soft-tissue lesions pre-operatively in order to plan surgery based on an accurate diagnosis.

Nowadays MRI is increasingly used in veterinary medicine to visualize various pathologies. Only a few authors have described high-field MRI for the diagnosis of intrathecal tendon lesions so far [11,12,13,14]. While 3 Tesla MRI has a very high diagnostic performance for soft-tissue imaging, general anaesthesia is necessary, and it remains logistically cumbersome and time-consuming. When considering standing diagnostic imaging technologies, low-field MRI is a sought-after imaging modality for diagnosing DTFS pathologies. T2 sequences are necessary to achieve hypointense soft tissue to intrathecally hyperintense fluid contrast. In terms of spatial resolution and anatomical detail, T1 sequences are considered superior. Injecting a contrast media intrathecally and acquiring post-contrast T1 sequences would combine the advantages of both and may improve the diagnostic performance of standing low-field MRI when diagnosing intrathecally situated soft-tissue lesions.

Gadolinium-enhanced MRI techniques have been used in human medicine for many years [15,16,17,18,19,20,21,22] and have also recently been tested and applied in veterinary medicine [23,24,25]. Administering gadolinium-based contrast media intrathecally results in the increased filling of the tendon sheath, outlining intra-synovial structures, and ultimately diffusion of contrast media into tears and depicting them [15,18,20,22,26]. The benefits of a direct MR arthrography of the canine elbow and stifle, as well as the equine distal interphalangeal joint, have been reported [27,28,29,30,31].

The objective of this study was to determine and compare the sensitivity and specificity of low-field MRI and direct low-field MRI DFTS tenography (MRIt) with gadolinium-based contrast media to detect artificially created lesions in the SDFT, DDFT and MF, as well as the proximal scutum. It was hypothesized that intrathecal gadolinium administration would improve the detection of intrathecally situated soft-tissue lesions compared to pre-contrast T1 and T2 sequences.

## 2. Materials and Methods

### 2.1. Specimens

Twenty-five distal limbs were obtained within 24 h after euthanasia (13 forelimbs and 12 hindlimbs) from Warmblood horses, for reasons unrelated to this study. Following the owners’ consent, the distal limbs were removed at the levels of the carpus and tarsus and stored at −28 °C. Prior to tenoscopy, the cadavers were defrosted in water until they reached room temperature (20 °C), then clipped and prepared for DFTS tenoscopy.

### 2.2. Tenoscopic Procedure

**Figure 1:**
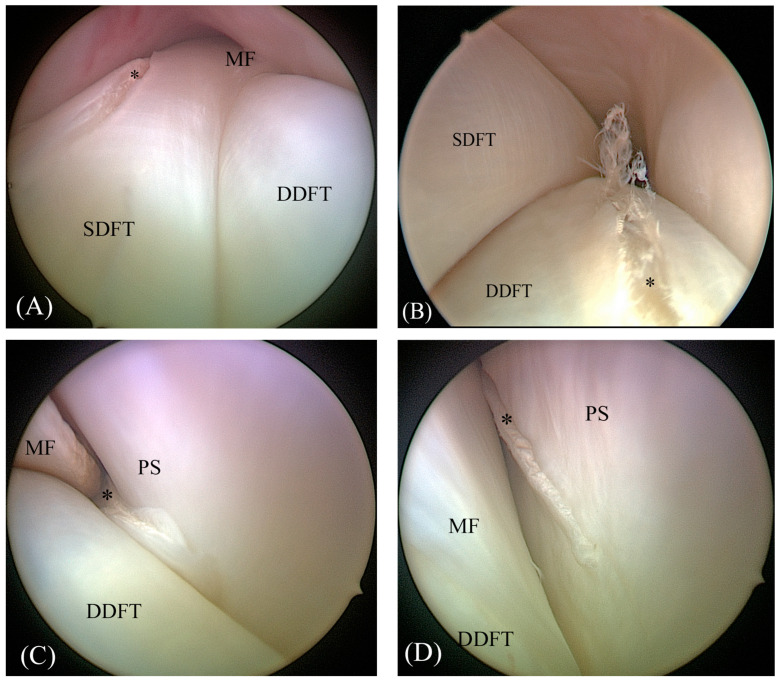
Images of tenoscopically created lesions (asterisk) in the superficial digital flexor tendon (SDFT) (**A**), the deep digital flexor tendon (DDFT) (**B**), the manica flexoria (MF) (**C**) and the proximal scutum (PS) (**D**).

A tenoscopy of the DFTS was performed by a standard medial or lateral (randomly determined by flipping a coin) approach by a 3rd-year surgical resident (AA) using a 30° forward angled, 4 mm rigid arthroscope (Karl Storz GmbH & Co. KG, Tuttlingen, Germany). Briefly, the distal pouch of the DFTS was distended with 40 mL NaCl; a standard arthroscopic portal was made into the DFTS, outpouching between the palmar annular ligament and the proximal digital annular ligament. The skin incision was made slightly palmar/plantar to the centre of the outpouching and 3–6 mm palmar/plantar to the neurovascular bundle. The arthroscopic sleeve was introduced using a conical obturator, and the sleeve was directed proximad through the fetlock canal into the proximal recess of the DFTS [32]. As a first step, the SDFT, DDFT, MF and proximal scutum were examined for preexisting lesions. Only limbs without lesions were included in this study. Then, an instrument portal was made in the proximal recess ipsilateral to the arthroscopic portal in a standard fashion. Using an arthroscopic hook knife (blade dimensions: 3 mm long and 1 mm wide), lesions in all the structures were randomly created (Figure 1). For the SDFT, DDFT and proximal scutum, one or two lesions (small and/or large) were placed, or the structure was left intact, serving as a negative control. The lesions (or negative controls), randomly determined by flipping a coin, were made on the ipsilateral side of the arthroscopic portal (medial or lateral), and the structures on the contralateral side always served as negative controls. Lesion depth in the SDFT, DDFT and proximal scutum was standardized to 3–5 mm based on the dimensions of the hook knife. The defects were categorized according to their size: SDFT lesions as small (5–10 mm) or large (15–20 mm); DDFT and proximal scutum lesions as small (15–20 mm) or large (30–40 mm). The choice of lesion size was based on the results of a previously performed experimental study, which showed inferior detection rates of DDFT lesions compared to SDFT and proximal scutum lesions using 3 tesla MRI [33]. Every tendon lesion was made by cutting into the structure parallel to its fibre course, creating small partial thickness lesions and fibre tearing parallel to the fibre course. DDFT and SDFT border lesions were located either distal to the MF or at the same height. This position was also randomly determined by flipping a coin.

MF lesions were defined as partial lesions. These partial lesions were like proximal scutum lesions cut perpendicular to their normal fibre course [4,34]. Partial lesions were defined as cutting half or less of the lateral or medial distal tendinous MF. Following tenoscopy, an 18-gauge, 5.1 cm catheter was placed under visual control into the proximal pouch on the contralateral side of the instrument portal, sutured in place and secured with tape. All the arthroscopic portals were closed, including the DFTS capsule and the skin. Following tenoscopy, all the limbs were stored at −28 °C.

### 2.3. Diagnostic Imaging

All the limbs were defrosted for 24 h before the diagnostic imaging procedure. MRI was performed with a 0.27 Tesla MRI scanner (Hallmarq Advanced Veterinary Imaging, Hallmarq Veterinary Imaging Ltd., Guildford, Surrey, UK). The limbs were positioned in a gantry and held in place by a custom-made wooden mount to achieve an upright position. Using a fetlock radiofrequency coil (Hallmarq Advanced Veterinary Imaging, Hallmarq Veterinary Imaging Ltd., Guildford, Surrey, UK), the DFTS of each limb was scanned. The MRI scanning protocol included a pre-contrast transverse T2-weighted fast spin echo motion insensitive (MI) and a transverse T1-weighted gradient echo MI sequence. Then, each DFTS was injected with 0.05 mL of gadolinium (0.5 mmol/mL) (Dotarem, Gadotersäure, Guerbet AG, Zürich, Switzerland), diluted with a 30 mL solution consisting of 10 mL iodinated contrast media (350 mg iodine/mL) (Ioversol, Optiray 350, Mallinckrodt AG, Steinhausen, Switzerland) and 20 mL of 0.9% NaCl saline, which was injected through the pre-placed catheter. This was distributed within the DFTS by extending and flexing the limb. Post-contrast MRI scans consisted of a transverse T1-weighted gradient echo MI sequence. MRI sequences, planes and acquisition parameters are summarized in Table 1.

### 2.4. Image Analysis

Images were examined in all planes for the presence or absence of lesions in the aforementioned soft-tissue structures in the DFTS using a diagnostic workstation and medical imaging software (Intellispace PACS Radiology 4.4553.0, Phillips Healthcare, Zurich, Switzerland). All the images were examined by a 2nd-year large animal diagnostic imaging resident under the supervision of a diplomate radiologist (AB, SO), and a consensus was reached. The radiologists were blinded to the presence, location, severity and total number of lesions within each DFTS. The radiologists received a randomly generated order in which the images had to be reviewed. The radiologists were not allowed to compare the pre- and post-contrast images (MRIt), but the pre-contrast T1 and T2 sequences were evaluated together. Based on the current literature, the normal MRI appearance of the investigated soft-tissue structures was defined [13]. A lesion was considered present if contrast media/synovial fluid was infiltrating with a linear or focal contrast accumulation/hyperintense signal in the respective normal hypointense structure.

### 2.5. Statistical Analysis

Data were stored in Microsoft Excel. The normal distribution of the data for continuous variables was assessed using the Kolmogorov–Smirnoff test. Parametric data were reported as the mean ± standard deviation, and non-parametric data were reported as the median (range).

For all the lesions of the different structures, the sensitivity and specificity of MRI and MRIt were calculated. Tenoscopy served as the reference. Sensitivity and specificity values were compared between all the modalities using McNemar’s tests. Binary logistic regression was used to evaluate the association of the soft-tissue structure (SDFT, DDFT, MF or proximal scutum), contrast administration (yes, no) and lesion length (mm) on the MRI and MRIt diagnoses (no lesion, lesion present). All the statistical analyses were performed with a commercially available statistical software program (R Core Team (2016). R: A language and environment for statistical computing. R Foundation for Statistical Computing, Vienna, Austria. URL https://www.R-project.org/ (accessed on 1 January 2023).) using the MASS, car and lme4 packages. A *p*-value < 0.05 was considered statistically significant.

## 3. Results

A total of 13 forelimbs (7 left and 6 right) and 12 hindlimbs (6 left and 6 right) of 7 cadaveric middle-aged Warmblood horses of different genders underwent DFTS tenoscopy. Four limbs had to be excluded from this study due to technical issues in the imaging part of the study (air artefacts or insufficient contrast filling). A total number of 118 lesions were created in 21 DFTSs. In each DFTS, a median of five lesions (four–seven lesions) were made.

The median lesion size in the SDFT, DDFT and proximal scutum was 10 mm (range: 5–30 mm).

The quality of the MRI and MRIt studies was considered good in all the included DFTSs. However, in all the DFTSs, a mild extravasation of contrast media/synovial fluid was visible, with both modalities following the arthroscopic portals.

**Figure 2:**
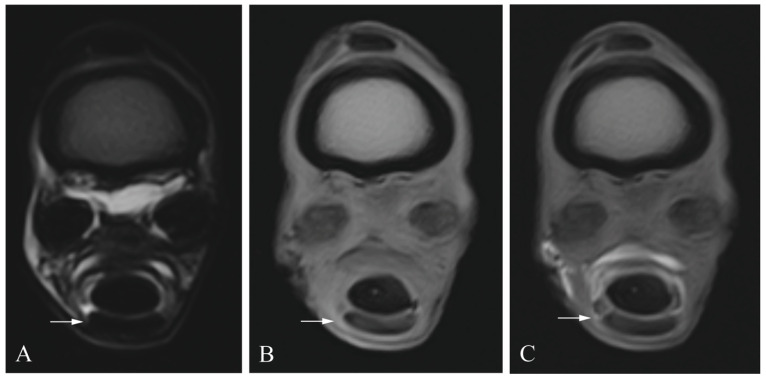
Superficial digital flexor tendon lesion (arrow) in a cadaveric equine digital flexor tendon sheath: transverse T2 magnetic resonance image (**A**), transverse T1 magnetic resonance tenographic image (**B**) and a transverse post-contrast T1 magnetic resonance tenographic image (**C**) of the same limb.

**Figure 3:**
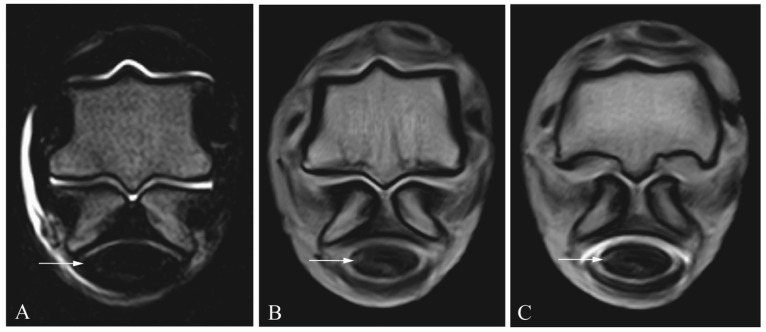
Deep digital flexor tendon lesion (arrow) in a cadaveric equine digital flexor tendon sheath: transverse T2 magnetic resonance image (**A**), transverse T1 magnetic resonance tenographic image (**B**) and a transverse post-contrast T1 magnetic resonance tenographic image (**C**) of the same limb.

**Figure 4:**
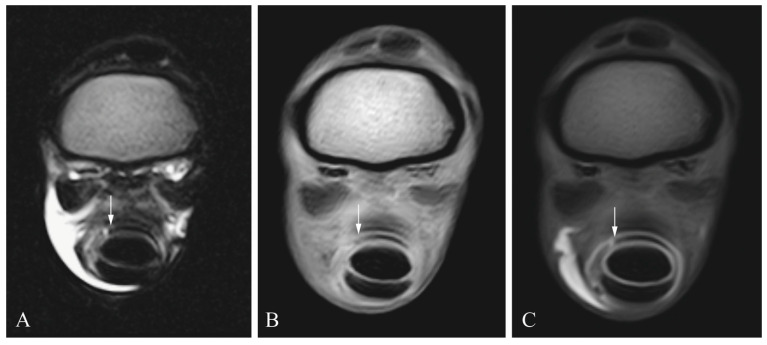
Manica flexoria lesion (arrow) in a cadaveric equine digital flexor tendon sheath: transverse T2 magnetic resonance image (**A**), transverse T1 magnetic resonance tenographic image (**B**) and a transverse post-contrast T1 magnetic resonance tenographic image (**C**) of the same limb.

**Figure 5:**
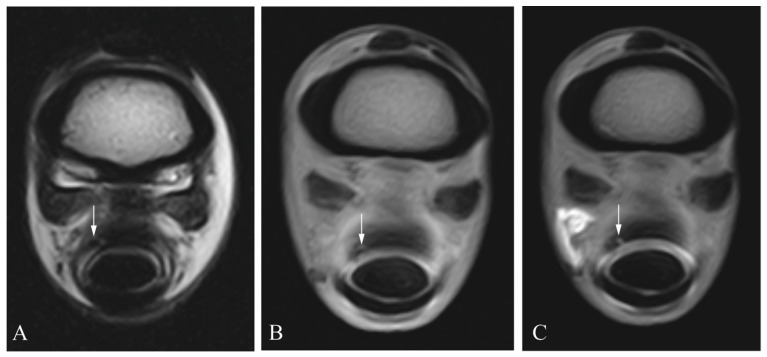
Proximal scutum lesion (arrow) in a cadaveric equine digital flexor tendon sheath: transverse T2 magnetic resonance image (**A**), transverse T1 magnetic resonance tenographic image (**B**) and a transverse post-contrast T1 magnetic resonance tenographic image (**C**) of the same limb.

Sensitivity and specificity values for the different soft-tissue structures are shown in Table 1. Figure 2, Figure 3, Figure 4 and Figure 5 show the different lesions visible on the MRI and MRIt. Briefly, MRI and MRIt showed similar sensitivity and specificity values for lesions in the SDFT (MRI (39% sensitivity and 93% specificity) in comparison to MRIt (54% sensitivity, *p* = 0.72; and 96% specificity, *p* = 1)). For proximal scutum lesions, sensitivity and specificity were the same for MRI and MRIt (38% sensitivity and 50%, respectively, *p* = 0.80; 96% specificity and 96%, respectively, *p* = 1). There was also no statistically significant difference between MRI (34% sensitivity; 100% specificity) and MRIt (32% sensitivity, *p* = 0.77; 98% specificity, *p* = 1) for DDFT lesions. MF lesions were detected by MRI with 61% sensitivity, which was also comparable to MRIt (50% sensitivity, *p* = 0.68). The specificity values (96%) were equally high for MF lesions diagnosed by MRI and MRIt (*p* = 1).

The confidence intervals were fairly large for the sensitivity values (0.18–0.83) and narrower for the specificity values (0.83–1) (Table 2). Neither lesion site nor gadolinium administration into the DFTS was associated with making a correct MRI or MRIt diagnosis (*p* = 0.47–0.79). Only lesion size was significantly associated with making a correct MRI or MRIt diagnosis (*p* = 0.001).

## 4. Discussion

The pre-operative diagnosis of DFTS pathology is always a challenging task, and, therefore, tenoscopy is considered the gold standard for diagnosing intrathecally located soft-tissue lesions. However, 3D imaging modalities have a very high diagnostic potential and could represent a step forward in the diagnosis of DFTS pathologies pre-operatively, especially when performed in a standing set-up. Hence, this study aimed to determine the diagnostic value of low-field MRI and direct MRIt for the detection of artificially created soft-tissue lesions within the DFTS. The authors hypothesized that MRIt would improve the detection rate of intrathecally located soft-tissue lesions compared with MRI. This hypothesis was refuted in this study, as intrathecal gadolinium administration did not improve the diagnostic performance of low-field MRI.

T1, in contrast to T2 sequences, have better anatomical detail and are less susceptible to motion artefacts, which, when combined with the administration of gadolinium, may make these sequences a better alternative for investigating tendon lesions within the DFTS. Gadolinium is a paramagnetic contrast media and causes a shortening of both T2 and T1 relaxation times, but changes in T1 relaxation times are dominant, resulting in increased signal intensity from the enhanced tissues or fluids in images acquired with rapid repetition [35]. The T1-weighted signal intensity of gadolinium depends on its concentration and magnetic field strength [15]. Despite the theoretical advantages of the T1 sequence in combination with gadolinium injection over the T2 sequence, there was no statistical difference between pre- and post-contrast MRI studies in their ability to visualize tendon lesions within the DFTS in this study.

Looking at the plain MRI sequences, radiologists used the T2 sequence more often than the pre-contrast T1 sequence to make a diagnosis in this study. Lesions were easier to detect in the T2 sequence, as is shown for proximal scutum lesions in Figure 5. This is due to the well-known fact that, in the T2 sequence, there is a better contrast between the hyperintense fluid within the tendon sheath and the hypointense tendon; therefore, defects of the tendon’s surface or hyperintense signal within a tendon are better detected as compared to the pre-contrast T1 sequence.

Regarding the sensitivity and specificity values, MRIt showed slightly better results for SDFT and proximal scutum lesions, whereas MRI, in comparison to MRIt, was better at detecting DDFT and MF lesions. Whether these small differences between modalities could become statistically significant in a larger number of cases remains to be confirmed.

Comparing the results of this study with a similar study using 3 tesla MRI to diagnose artificial lesions within the DFTS, both low-field MRI and MRIt achieved lower sensitivity values for each structure [33]. This is an expected finding. The main disadvantage of low-field compared to high-field MRI is its inferior signal to noise ratio and lower spatial resolution [36]. Low-field MRI produces images with a much greater slice thickness, which is an additional disadvantage [37,38]. Slice thickness likely contributes significantly to the low sensitivity results of low-field MRI and MRIt for the rather small artificial tendon lesions created in this study. The median lesion length in our study was 10 mm (range: 5–30 mm). Depending on the sequence used, the slice thickness was 5 mm (see Table 1). Looking at these values, it is easy to explain that small lesions of 5–10 mm were easily missed or simply not displayed, due to volume averaging. The binary logistic regression analysis of lesion length in our study supports this finding, as lesion length had a significant impact on whether a correct MRI diagnosis was made or not, independent of the use of contrast media. Volume averaging may play an important role as to why small lesions were not detected or were measured as significantly smaller. Also, the normally hyperintense contrast appears rather grey in certain areas, which can lead to defects being overlooked [39]. In this study, we placed artificial lesions that were, of course, not directly comparable to naturally occurring lesions. Fibrillations and general secondary changes within the DFTS as present in a clinical scenario were missing, and could not help the radiologists in identifying the lesions. In addition, it should be noted that naturally occurring longitudinal tendon tears are usually much larger than the lesions placed in this study. Large lesions have previously been classified as over 7 cm long, and small lesions have been classified as under 7 cm long [1]. The lesions placed here were considerably smaller, with a maximum length of 3 cm. The lesions created in the study should test the limits of the modality. Hence, it is expected that MRI would achieve higher sensitivity and specificity values for the detection of naturally occurring lesions; however, this remains to be confirmed.

To date, there is no established standard protocol for the concentrations at which gadolinium should be used in small animals or horses for various synovial structures yet. In humans, gadolinium concentrations of 2–5 mmol/L have been described for intra-articular use, with 2 mmol/L being the most commonly used concentration for a wide variety of applications [22,40,41,42]. Others report optimal gadolinium concentrations of 0.7–3.5 mmol/L [43]. There are studies in small animals that used lower gadolinium concentrations and achieved good detection rates of shoulder pathologies; and another study, which tested different gadolinium concentrations, showed that lower concentrations were also diagnostic [27,44,45]. A maximum contrast signal intensity is not always advantageous, as it may obscure mild to moderate changes in the lesion periphery [45]. This observation is supported by several in vitro studies showing that the optimal gadolinium concentration significantly shifts to lower concentrations when mixed with iodine. In this study, we used a final gadolinium concentration of 0.75 mmol/L and injected with it iodine at a concentration of 117 mg/mL. These concentrations are within the previously described ranges, where gadolinium concentrations of 0.625–1.25 mmol/L in combination with 30–300 mg iodine/mL have been recommended [43,46,47]. Iodine was injected into the tendon sheath, and these concentrations were chosen because the same specimens were used in a master’s thesis study.

The tenoscopic portals could not be completely sealed, despite multiple-layer closure. As a result, in some cases, after injecting a volume of 30 mL, contrast media leaked into the subcutaneous tissue surrounding the tenoscopic portals, causing hyperintensity in this area. In humans, diagnostic difficulties due to extraarticular injection or contrast media leakage through the capsular puncture site are also known [15]. This problem may make it difficult to identify individual lesions. The leakage and resulting artefacts affected the pre contrast images equally in this study and should not have led to a difference between MRI and MRIt. However, they may have contributed to the overall low results of both.

Specificity values for all lesion types were very high for both MRI (93–100%) and MRItt (96–98%). They appear to be highly accurate for the diagnosis of a normal, i.e., intact, soft-tissue structure.

The limitations of the study are the small number of cadaver limbs used. This is a cadaver study, and the artificial lesions placed here cannot be directly compared with naturally occurring marginal tendon lesions. Most importantly, lesions in a natural setting are usually larger than those created in this study. In addition, secondary changes are common in natural lesions and extremely helpful for making a diagnosis. Another limitation, as mentioned above, is that the tenoscopic portals could not be completely sealed and, as a result, contrast media leakage occurred at the portals using a volume of only 30 mL. Furthermore, there are still no studies that have determined the optimal volume for the tenography of DFTS pathologies. Whilst injecting contrast media into intact DFTS in clinical patients, contrast leakage into the surrounding tissue will occur to a lesser extent, and much higher volumes and pressures should be achieved in clinical cases, potentially increasing the diagnostic performance of MRIt. As only one radiologist was available to review the MRI and MRIt studies, we were unable to assess the interobserver agreement and reliability of our data. To avoid creating multiple arthroscopy portals where contrast extravasation could occur, all the lesions were created ipsilateral to the tenoscopy portals. Of course, the tenoscopy portals were visible in both MRI and MRIt. Although the radiologist was blinded and had to recognise a lesion against a negative control, this may have led to a minor bias of the radiologists over time, as they may have had a learning curve. However, this bias is similar for MRI and MRIt, and should not have led to a difference in the comparison between both. Overall, the radiologists still had to detect the lesions in each structure or deem a structure as healthy. We only investigated whether the use of contrast in the DFTS made a difference in the diagnosis of soft-tissue lesions. It was not objectively assessed whether the pre-contrast T1 or T2 sequence was better for making a diagnosis, which is another limitation of this study. Furthermore, adding high resolution sequences with lower slice thickness may have improved the pre contrast diagnostic performance compared to the protocol used.

## 5. Conclusions

MRIt did not improve the diagnostic performance of low-field MRI to detect artificially created tendon lesions within the DFTS. From the results of this study, the intrathecal administration of gadolinium cannot be recommended to improve the diagnostic performance of low-field MRI examination of the DFTS. However, whether this also applies to clinical cases with naturally occurring, intrathecally situated tendon lesions remains to be proven. The specificity of both MRI and MRIt was high, thus being accurate in diagnosing an intact soft-tissue structure. Small lesion length was a significant discriminating factor for the likelihood of lesion detection. These are important results for the future use of low-field MRI in clinical cases, for instance when aiming to exclude certain intrathecally situated soft-tissue lesions in horses without them needing to undergo tenoscopy.

## Figures and Tables

**Table 1:** Scanning parameters of the magnetic resonance imaging sequences used in this study. Abbreviations: FOV: field of view, TR: time to repetition, TE: time to echo, NEX: number of excitations, SNR: signal to noise ratio, TRA: transverse, GRE: gradient echo, MI: Motion insensitive, FSE: fast spin echo.

	T2W_FSE_TRA_MI Pre-Contrast	T1W_GRE_TRA_MI Pre-Contrast	T1W_GRE_TRA_MI Post-Contrast
TR	1544	52	52
TE	88	8	8
Flip angle	90	50	50
Echo	8	1	1
NEX	1	1	1
FOV	170	171	171
Slice thickness	5 mm	5 mm	5 mm
Gap width	1 mm	1 mm	1 mm
Number of slices	8	8	8
Time to run (min)	2:10	2:10	2:10
SNR	94	99	99
Spacing between slices	6.0	6.0	6.0

**Table 2:** Sensitivity and specificity of magnetic resonance imaging (MRI) and magnetic resonance imaging tenography (MRIt) to detect experimental lesions in different soft-tissue structures within the digital flexor tendon sheath.

Lesion	Sensitivity (%) (95% ^), (*n*)	Specificity (%) (95% ^), (*n*)
MRI SDFT	39% (0.22–0.59) (11)	93% (0.83–0.98) (52)
MRIt SDFT	54% (0.34–0.72) (15)	96% (0.88–0.99) (54)
MRI DDFT	34% (0.19–0.51) (13)	100% (0.92–1) (46)
MRIt DDFT	32% (0.18–0.49) (12)	98% (0.88–0.99) (45)
MRI MF	61% (0.36–0.83) (11)	96% (0.79–0.99) (23)
MRIt MF	50% (0.26–0.74) (9)	96% (0.79–0.99) (23)
MRI scutum	38% (0.22–0.56) (13)	96% (0.86–0.99) (48)
MRIt scutum	50% (0.32–0.68) (17)	96% (0.86–0.99) (48)

## Data Availability

Data contained within the article.
